# Seroprevalence and Virologic Surveillance of Enterovirus 71 and Coxsackievirus A6, United Kingdom, 2006–2017

**DOI:** 10.3201/eid2709.204915

**Published:** 2021-09

**Authors:** Everlyn Kamau, Dung Nguyen, Cristina Celma, Soile Blomqvist, Peter Horby, Peter Simmonds, Heli Harvala

**Affiliations:** University of Oxford, Oxford, UK (E. Kamau, D. Nguyen, P. Horby, P. Simmonds);; Public Health England, London, UK (C. Celma);; National Institute for Health and Welfare, Helsinki, Finland (S. Blomqvist);; National Health Service Blood and Transplant, London, UK (H. Harvala); University College London, London, UK (H. Harvala)

**Keywords:** hand foot and mouth disease, enterovirus, EV-A71, CVA6, seroprevalence, viruses, antibodies, immunity, exposure, United Kingdom, England, coxsackievirus

## Abstract

Enterovirus A71 (EV-A71) and coxsackievirus A6 (CVA6) cause hand, foot and mouth disease (HFMD) and are occasionally linked to severe neurologic complications and large outbreaks worldwide. We estimated EV-A71 and CVA6 seroprevalence using cross-sectional age-stratified samples collected in 2006, 2011, and 2017. Seroprevalences of EV-A71 and CVA6 increased from 32% and 54% at 6–11 months to >75% by 10 years of age. Antibody titers declined after 20 years, which could indicate infrequent re-exposure in older populations. Age profiles for acquiring infections and mean titers were comparable in the 3 testing years, despite the marked increase in incidence of CVA6-related HFMD from 2010. The uncoupling of changes in disease severity from the infection kinetics of CVA6 as we inferred from the seroprevalence data, rather than incidence of infection over the 11-year study period, provides further evidence for a change in its pathogenicity.

Enteroviruses within species A are the primary cause of hand, foot and mouth disease (HFMD), mostly affecting infants and young children. HFMD is highly contagious and manifests as a self-limiting illness; it typically includes fever, skin eruptions on hands and feet, and vesicles in the mouth (*1*,*2*). In severe disease, patients develop neurologic and systemic complications that can be fatal, including meningoencephalitis, pulmonary edema, and acute flaccid paralysis (*3*,*4*).

Enterovirus A71 (EV-A71) is the predominant cause of HFMD outbreaks. In the Asia-Pacific region, the effects of the virus on public health have been substantial; in Europe these infections are considered mild and often remain undiagnosed (*5*), although severe neurologic manifestations and small outbreaks have been reported more recently (*6*–*10*). EV-A71 is classified into 7 genogroups (A–G) and several subgenogroups (B0–B5, C1–C5) based on the viral protein 1 gene; the appearance of novel EV-A71 genogroups has been associated with large HFMD outbreaks (*5*).

Coxsackievirus A6 (CVA6) has become another major cause of HFMD since 2008 (*11*,*12*). CVA6 infections have often been linked to a febrile atypical form of HFMD, affecting both pediatric and adult populations (*13*–*15*). The severity of the clinical manifestations associated with CVA6 infections and the recent increase of HFMD cases associated with EV-A71 and CVA6 in Europe (*10*) may have originated through the evolution of recombinant forms or changes in pathogenicity of emerging strains (*16*,*17*). Alternatively, their clinical prominence may have resulted from an increase in infections in a larger previously unexposed and susceptible populations. To investigate that theory, we determined the age-stratified seroprevalence of EV-A71 and CVA6 in representative cross-sections of the UK population in 2006, 2011, and 2017; we used serotype-specific microneutralization assays and compared our findings with the numbers of infections reported through public health surveillance.

The 2011 timepoint corresponded to the approximate timing of large EV-A71 outbreaks, especially in Vietnam and China (*12*,*18*) in addition to emergence of CVA6 infections associated with atypical clinical phenotypes (*11*,*19*). Whereas the 2006 timepoint was selected to precede these recorded events and the 2017 to measure population immunity post-CVA6 emergence period, the last timepoint also corresponded to recorded EV-A71 outbreaks in Spain and elsewhere in Europe in 2016 (*4*,*7*,*8*). Collectively, these selected timepoints reflected changed activity of both viruses and hence enabled us to measure their effects on population immunity.

## Materials and Methods

### Serum Samples

We obtained a convenience sample of 1,573 residual serum samples collected in 2006 (n = 514), 2011 (n = 498), and 2017 (n = 561) from the seroepidemiology unit archive collection of Public Health England (PHE; Manchester, UK). This archive is an opportunistic collection of residual clinical samples from laboratories throughout England. Case-patients were divided into 7 age groups: <6 months, 6–11 months, 1–5 years, 6–10 years, 11–20 years, 21–40 years, and >40 years. We aimed to obtain 100 samples from each group (Appendix). We anonymized all samples and unlinked any patient identifying information; we retained age, sex, date of collection, sample type, and contributing laboratory information.

### Virus Strains

We obtained 2 CVA6 strains isolated in Finland in 2008 and 2016 from the National Institute for Health and Welfare (Helsinki, Finland). The CVA6/2008 isolate was obtained during a HFMD outbreak in Finland (*20*), and the CVA6/2016 isolate was a contemporary clinical strain. We used the EV-A71 genogroup B4 strain isolated in Singapore (5865/SIN/000009). We propagated EV-A71 viruses in a rhabdomyosarcoma cell line obtained from the American Type Culture Collection. We propagated CVA6 viruses in TE32 or 130T cells obtained from the UK National Institute for Biologic Standards and Control. We determined the 50% tissue culture infective dose (TCID_50_) of virus stocks by means of endpoint dilution using the Reed and Muench method: in a 96-well format, 8 replicates of a 10-fold serial dilution were incubated with cells in Dulbecco minimum essential medium (DMEM; Sigma-Aldrich, https://www.sigmaaldrich.com) containing 2% vol/vol fetal bovine serum (FBS; Sigma-Aldrich) and penicillin/streptomycin (10,000 U/mL; Sigma-Aldrich) at 37 °C in 5% CO_2_ for 4–5 days.

### Neutralization Assays

The microneutralization assay was performed as previously described (*21*) (Appendix). In brief, we inactivated serum samples for 3 min at 56°C, and then diluted 2-fold serially in 2% DMEM-FBS from 1:8 to 1:1,024. We mixed 50µL of diluted samples and 100 TCID_50_ of virus stock diluted in 50 µL in 96-well microplates and incubated at 37°C for 1 hour. We added 100 µL of cell suspension containing average of 20,000 rhabdomyosarcoma cells in 10% DMEM-FBS for EV-A71 assays and average of 20,000 TE32 cells in 5% DMEM-FBS for CVA6 assays. We observed cytopathic effect in an inverted microscope after incubating at 37°C in 5% CO_2_ for 4–5 days. We used pooled adult serum with known neutralizing antibody titer (nAb; 13/328, obtained from the UK National Institute for Biologic Standards and Control) as a positive control and inactivated horse serum (obtained from American Type Culture Collection) as negative control. We included a virus control and an uninfected cell control for each batch of tests. We tested each sample in duplicate and calculated results as their geometric mean titers (GMT).

**Figure 1:**
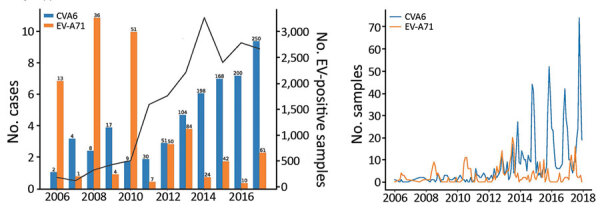
EV-A71 and CVA6 identified in enterovirus-positive samples referred to Public Health England from laboratories throughout England, UK, by year, 2006–2017. A) Percentage of samples typed as EV-A71 and CVA6 in each referral year (total no. cases above each bar). Solid black line indicates number of samples referred for virus typing. B) Distribution of EV-A71 (n = 381) and CVA6 (n = 1,033) clinical detections in England, using monthly totals for the period 2006–2017. CVA6, coxsackievirus A6; EV, enterovirus; EV-A71, enterovirus A71.

To determine the optimal strain for the CVA6 neutralizing assay, we compared titers of 36 serum samples collected in 2006 against the 2 CVA6 clinical isolates. We selected 18 samples each for the 1–5-year (representing serologic responses acquired during 2001–2006) and >40-year (representing serologic responses acquired substantially before 2006) age groups. For the 1–5-year age group, 16/18 samples tested were seropositive for the CVA6/2008 and 17/18 samples tested were seropositive for CVA6/2016 isolates. All 18 samples tested from the >40-year age group were seropositive for both CVA6 isolates. GMT to both CVA6 isolates were comparable between the 1–5-year and >40-year age groups (Appendix, Figure 1). Samples collected from the >40-year age group in 2006 had proportionately higher nAb against the CVA6/2008 isolate (p = 0.008 by paired Wilcoxon signed rank test). Because the differences in GMT between the CVA6 isolates were minor, we selected the more contemporary CVA6/2016 strain for the assay used in this study.

We reported the neutralizing titer as the reciprocal titer of serum dilutions that inhibited 50% virus growth. For both EV-A71 and CVA6, samples with nAb titers of >1:8 were considered seropositive as previously reported (*22**,**23*). For GMT calculations, we excluded titers <1:8; we assigned a value of 2,048 to titers >1:1,024. We classified GMT values as low (<1:64), moderate (1:64–1:256), and high (>1:512).

### Virological Surveillance Data

We collected information on enterovirus-positive samples submitted for typing to the PHE Enteric Virus Unit (London, UK), during 2006–2017. Local diagnostic laboratories in England and Wales were asked to forward samples in which EV RNA has been detected for typing, for the purposes of national enhanced enterovirus surveillance. Data collected included a total number of enterovirus-positive samples submitted for typing and the number identified as EV-A71 or CVA6 per month, patient age group, and sample type.

We used these data to compare the prevalence of infections estimated from serologic data with EV-A71– and CVA6-associated infections reported through this voluntary enhanced enterovirus surveillance.

### Statistical Analysis

We compared rates of seropositivity in different groups using χ^2^ or Fisher exact test, with Bonferroni adjustment for multiple comparison. We compared age-stratified GMTs between the serum collection time points using the Mann-Whitney U or Kruskal-Wallis test with Dunn’s post hoc analysis. We calculated 95% CIs of the seroprevalence rates according to the Wilson method (http://vassarstats.net/prop1.html) and considered p<0.05 statistically significant. We computed all the statistical analyses in R (https://www.r-project.org).

## Results

### Enterovirus Reporting in the United Kingdom, 2006–2017

We identified 402 EV-A71–positive and 1,519 CVA6-positive samples from 20,221 enterovirus-positive samples referred to PHE for typing (Figure 1, panel A). Over the study period, the numbers of enterovirus-positive samples referred for typing increased substantially from 189 in 2006 to 1,479 in 2017. At the same time, the proportion of samples typed as CVA6 increased sharply, from ≈1% in 2007–2008 to 10% in 2016–2017, and the proportion of samples typed as EV-A71 decreased.

Most EV-A71 infections were reported in even years; ≈10% of all enterovirus-positive samples were identified as EV-A71 in 2006, 2008, and 2010, whereas this proportion has remained at ≈3% since 2012. The peak months for EV-A71 detections were July–August and for CVA6 detections were October–December. The highest monthly detections were 20 of EV-A71 in July 2013 and 74 of CVA6 in October 2017 (Figure 1, panel B).

EV-A71 infections were mostly identified in feces (122/381, 32%; data not available for 21 samples), followed by cerebrospinal fluid (CSF) (100/381, 26.2%), respiratory specimens (46/381, 12.1%), vesicle or skin swabs (21/381, 5.5%), and blood (24/381, 6.3%) (Table). Consistent with its association with HFMD in the UK, CVA6 was mostly detected in vesicle or skin swabs (759/1,033, 73.5%; data not available for 486 samples), followed by respiratory specimens (136/1,033, 13.2%), feces (84/1,033, 8.1%), CSF (44/1,033, 4.3%), and blood (42/1,033, 4.1%).

**Table:** EV-A71– and CVA6-positive samples submitted to the Public Health England Enteric Virus Reference Department, United Kingdom, 2006–2017*

Virus	Blood	CSF	Gastrointestinal	Respiratory	Skin	Tissue	Total
EV-A71	24 (6.3)	100 (26.2)	122 (32)	46 (12.1)	21 (5.5)	25 (6.6)	381
CVA6	42 (4.1)	44 (4.3)	84 (8.1)	136 (13.2)	759 (73.4)	19 (1.8)	1,084

*Totals of positive samples are given as no. (%). CSF, cerebrospinal fluid; CVA6, coxsackievirus A6; EV-A71, enterovirus A71.

**Figure 2:**
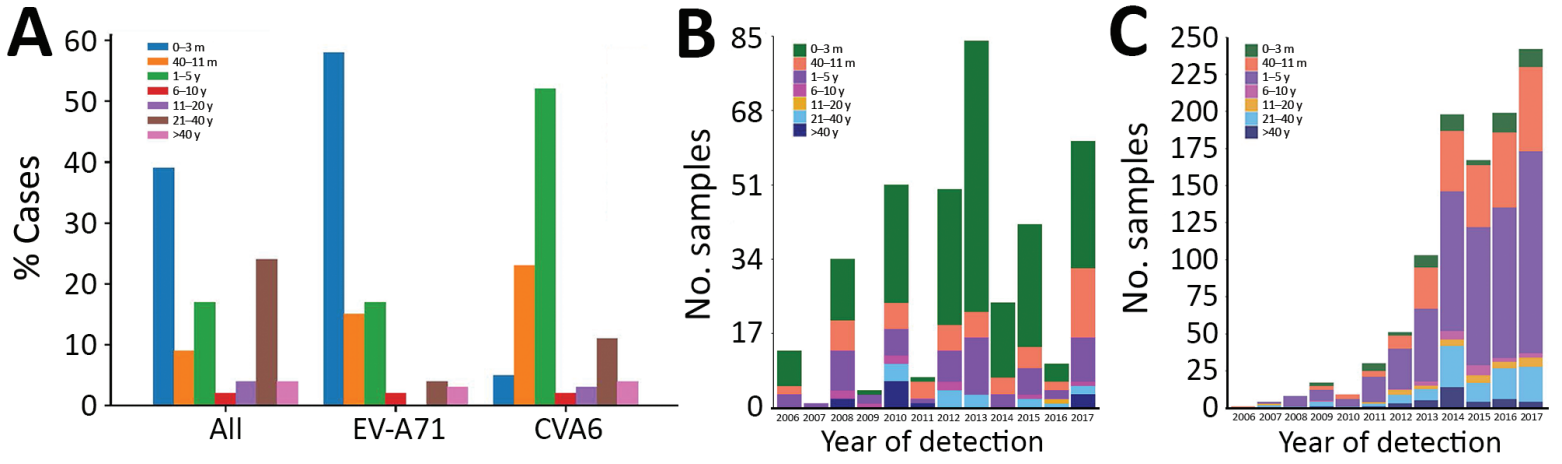
EV-A71 and CVA6 identified in enterovirus-positive samples referred to Public Health England from laboratories throughout England, UK, by age, 2006–2017. A) Percentage of all enterovirus-positive samples, by age group. B, C) EV-A71 (B) and CVA6 (C) detection by age group and by year of sampling. CVA6, coxsackievirus A6; EV-A71, enterovirus A71.

Patient age data were available for 9,636/20,211 total samples. Age data were available for 381/402 EV-A71 samples, and for 1,029/1,519 CVA6 samples. Most enterovirus-positive samples were obtained from young children <3 months of age (3,730/9,636, 39%), or young adults (2,309/9,636, 24%) (Figure 2, panel A). EV-A71 detections were highest in infants <3 months (222/381, 58%), whereas 58/381 (15%) were identified in children 4–12 months of age and 63/381 (17%) in children 1–5 years of age. CVA6 infections were diagnosed most often in older children 1–5 years of age (52%, 537/1,029), followed by children 4–12 months of age (23%, 239/1,029). In contrast, a small number of CVA6 infections were reported in infants <3 months of age (56/1,029, 5%). We observed no change in EV-A71 or CVA6 detection by age group for 2006–2017 (Figure 2, panels B and C).

### Seroprevalence of EV-A71

**Figure 3:**
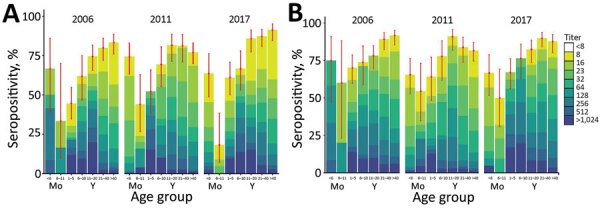
EV-A71(A) and CVA6 (B)seroprevalence in England, UK, in 2006, 2011, and 2017, by age group. Results are expressed as percentage of samples displaying neutralizing antibody titers <8 to >1,024 (colored bars). Red dots represent point estimates of the seropositive proportion; error bars indicate 95% CI. Samples were scored seropositive if neutralization was achieved at serum dilution of >1:8. CVA6, coxsackievirus A6; EV-A71, enterovirus A71.

The overall seropositivity rate of EV-A71 was 74% (95% CI 71.8%–76.2%). The seropositivity rates for the 3 timepoints were comparable at 71% (95% CI 66.8%–75.0%) in 2006, 73% (95% CI 69.1%–77.0%) in 2011, and 77% (95% CI 73.8%–80.9%) in 2017. Age-specific seroprevalence of EV-A71 nAb in each timepoint were lowest in children 6–11 months of age and gradually increased with age category (p<0.001 by χ^2^ test for trend) (Figure 3; Appendix Table 1). The seropositivity rate for the >40-year age group increased from 77% in 2011 to 91% in 2017 (p = 0.003 Fisher exact test).

The proportion of samples with moderate (64–256) and high (≥512) nAb titers increased with age from 1–20 years but decreased thereafter; most (>85%) samples from adults >20 years had titers <256 (Figure 3). For example, in 2006, the proportion of patients with high titers decreased from 30% in the 11–20-year age group to 6.7% in the 21–40-year age group and to 3.8% in the >40-year age group. We observed a similar trend of declining titers through 2011, in which the proportion of patients with high titers dropped by age group, from 12% (11–20 years) to 9% (21–40 years) to 2% (>40 years), and through 2017, when titers drop from 19% (11–20 years) to 11% (21–40 years) to 5% (>40 years).

The seropositive samples from infants (<6 months of age) in 2006 had a GMT 5-fold higher than the same age group in 2017, whereas those from children 6–11 months of age in 2006 had a geometric mean titer 3.6-fold higher than the same age group in 2017. Similarly, the samples from children 1–5 years of age in 2011 had a GMT 5.5-fold higher than in 2017 (Appendix, Table 1). Significant increases in titers of seropositive samples were found among children <6 months (p = 0.014 by Kruskal-Wallis test) and 1–5 years of age (p = 0.0026) and also among patients aged 11–20 years of age (p = 0.0067) (Appendix, Figure).

### Seroprevalence of CVA6

The seropositivity for CVA6 was 80% (95% CI 78.2–82.3) overall and 82% (95% CI 78.7–85.3) for 2006, 78% (95% CI 74–81.8) for 2011, and 80% (76.7–83.3) for 2017; seropositivity similarly increased with increasing age group (p<0.001 by χ^2^ test for trend) (Figure 3; Appendix, Table 2). The seropositivity rates were comparable across age groups (p>0.05 by Fisher exact test). We observed significant differences in CVA6 antibody titers among seropositive samples from children <6 months of age (p<0.001 by Kruskal-Wallis test), 1–5 years of age (p = 0.005), and 6–10 years of age (p<0.001). Neutralizing antibody titers were significantly lower in 2011 for seropositive samples (titer >8) in the 21–40-year and >40-year age groups (Appendix, Figure 2).

The proportion of infants <6 months of age with titers >64 was significantly higher in 2006 (75%) than in 2011 (17%) and 2017 (14%), whereas the proportion of adults >40 years of age with moderate titers was significantly lower in 2011 (27.8%) than in 2006 (49.2%) and 2017 (51.7%) (p<0.001 by Fisher exact test) (Figure 3). Geometric mean titers were highest in children 1–10 years of age in 2017 and >5-fold higher in 2006 for the <6-month-olds (Appendix Table 2).

## Discussion

Seroepidemiology findings in this study showed that EV-A71 and CVA6 infections were highly prevalent among children and adults in the United Kingdom. From the minimum values in the 6–11-month age group after the decline of maternally conferred immunity (*24**,**25*), we determined that EV-A71 and CVA6 neutralizing antibody detection frequencies and titers increased steadily with age, which indicates ongoing exposure and infection throughout childhood. EV-A71 seropositivity rates observed in the United Kingdom were comparable to those observed among preschool children <6 years of age (63.4%) in Germany (*26*) and in children <5 years of age in the Netherlands (*27*). EV-A71 seroprevalence in adults (>75%) was comparable for the United Kingdom, the Netherlands, and Germany.

The number of persons with high titers of EV-A71 neutralizing antibodies declined with age; this finding is consistent with previous seroepidemiological studies, including the report of high EV-A71 antibody titers in the 10–14-year age group in Germany (*28*), and comparable to the peak titers recorded in the 11–20-year age group in our study. These findings indicate that EV-A71 primarily circulates in and infects children, and the subsequent decline in titers but not frequencies of seropositivity indicates that re-exposure in the older population is uncommon (*28**–**30*). The decline in titers may also reflect the differences between acute serologic responses post-infection in the younger population and homeostatic antibody levels in the older population that become established years after infection (*30*). Related to this decline, the >4-fold attrition in mean EV-A71 neutralizing antibody titers in the 21–40-year age group (Appendix Table 1) may also create the low mean titers of maternally derived antibodies observed in children <6 months of age. This finding may underpin the high incidence of EV-A71 diagnosis reported in the 0–3-month age group when infants are most susceptible to severe infection outcomes (Figure 3). Of note, the largest share (39%) of enterovirus-positive samples were obtained from this age group, which might attest to infants’ vulnerability and higher likelihood of sampling.

The global emergence of CVA6 since 2008 has been linked to an increase in pathogenicity of CVA6 around 2010 (*31*), becoming another major causative agent for HFMD in several countries worldwide (*23*). This change was reflected in the number of atypical HFMD caused by CVA6 in Scotland in 2014 (*19*) and also in the increasing numbers of reported CVA6 infections in our study (Figure 1). Our seroprevalence data show that CVA6 circulated widely before the emergence of atypical HFMD in 2008 (*25*); seroprevalence approached 90% in adults >40 years of age as recorded in 2006 (Figure 3). This observation discounts the idea that the increased incidence of CVA6-associated HFMD simply reflects a change in its infection incidence and the existence of a widely susceptible population.

Comparing the 2 serotypes, CVA6 seroprevalence was higher than EV-A71 seroprevalence in younger children (1–10 years) in each study year (Figure 3; Appendix Tables 1, 2). However, this difference was not reflected in the peak age group for CVA6 infections (1–5 years) (Figure 2), which contrasts with the predominance of EV-A71 infections recorded in neonates and infants. CVA6 infections were predominantly detected in skin vesicle fluids (Table; Appendix), which would primarily be associated with HFMD manifestations (*32**–**34*).

Over the study period, the number of samples referred to PHE substantially increased (Figure 1), but rather than indicating more enterovirus-associated disease, this finding is more likely a reflection of improvements in detection through exclusive introduction of PCR in the clinical laboratories (*35*). Diagnostic practices in general, and for enteroviruses in particular, have changed over time in England and Wales as previously described (*35*). The use of PCR has increased rapidly, from 36% in 2000 to 45% in 2011, and probably approached 100% in 2015, replacing the slow and laborious virus culture entirely.

Changes in clinical practice or diagnostic procedures, such as the threshold for investigating and hospitalizing patients with suspected viral infections, or performing lumbar puncture (*35*), may have further influenced the number of samples submitted to PHE. Controlled cohort-based surveillance studies are required to better infer EV incidence.

A limitation of this study is that we based our inferences of incidences of EV-A71 and CVA6 infections on referral of clinical samples for typing at PHE. The much lower numbers of EV-A71–positive samples identified from older children and adults (Figure 2) at a time when seroprevalence was increasing (Figure 3) is indicative of subclinical infections or benign disease in these age groups. Differences in clinical practices could have also influenced the number of samples obtained and referred from older children and adults to PHE. For instance, CSF samples are more likely to be obtained for enterovirus testing from these patients who had any neurologic symptoms, compared with throat, fecal, or rectal swab specimens from which the viral loads would be higher and virus excretion prolonged (*36**,**37*). In addition, delayed lumbar puncture also reduces the likelihood of a positive pathogen detection. Atypical and varying clinical manifestations, especially in older adults, and the absence of CSF pleocytosis may also impede the timely diagnosis of enteroviral infections and consequently reduce the number of samples found to be positive and referred to PHE.

We used a convenience sample of residual serum samples from diagnostic laboratories throughout England. Although we attempted to include equal sample sizes for all ages, the serosurvey was not powered to provide precise seroprevalence estimates for certain age groups. The volume of available specimens, particularly for the younger age groups, was insufficient, thus limiting the number of samples tested and generalization of our results to the larger pediatric population. Convenience samples are also prone to chance variations in sampling between geographic regions. Lack of additional information on participants’ risk factors for exposure was another limitation.

In summary, we provide an analysis of age-stratified seroprevalence of EV-A71 and CVA6 in the UK population. Prevalence of infection by both viruses inferred from age-related changes in seroprevalence varied little over the 11-year study period despite the emergence of CVA6-associated HFMD in 2010, implying changes in CVA6 pathogenicity rather than changes in population susceptibility to severe infection outcomes. This study will enable a more detailed understanding of population susceptibility, the emergence of enterovirus serotypes, and potential changes in serotype pathogenicity and transmissibility.

Appendix Additional information about seroprevalence and virologic surveillance of enterovirus 71 and coxsackievirus A6, United Kingdom, 2006–2017.
