# Supplementary material for: Seroprevalence and Virologic Surveillance of Enterovirus 71 and Coxsackievirus A6, United Kingdom, 2006–2017
Source: Emerg Infect Dis. 2021 Sep;27(9):2261–8. doi: 10.3201/eid2709.204915 (PMC8386771; doi:10.3201/eid2709.204915)
Supplement: Appendix — Additional information about seroprevalence and virologic surveillance of enterovirus 71 and coxsackievirus A6, United Kingdom, 2006–2017. [file 20-4915-Techapp-s1.pdf]

Article DOI: https://doi.org/10.3201/eid2709.204915

Seroprevalence and Virologic Surveillance of Enterovirus 71 and Coxsackievirus A6, United Kingdom, 2006–2017

Appendix

Virus Isolates and Cell Lines

We obtained 2 coxsackievirus A6 (CVA6) strains isolated in 2008 and 2016 from the National Institute for Health and Welfare, Helsinki, Finland. The CVA6/2008 isolate was obtained during a HFMD outbreak in Finland, and the CVA6/2016 isolate was a contemporary clinical strain also obtained from Finland. We used the enterovirus strain EV-A71 genogroup B4 strain isolated in Singapore (5865/SIN/000009).

We propagated EV-A71 viruses in rhabdomyosarcoma cell line (RD) obtained from the American Type Culture Collection (ATCC). We propagated CVA6 viruses in TE32 or 130T cells obtained from the UK National Institute for Biologic Standards and Control.

The RD cells were maintained in Dulbecco's Minimum Essential Medium (DMEM/F-12, Gibco) containing 10% v/v fetal bovine serum (FBS, Sigma-Aldrich) and penicillin–streptomycin (Sigma-Aldrich, 10,000 U/mL) at 37°C, 5% CO_2. The TE32 or 130T cells were maintained in DMEM containing 5% v/v FBS and penicillin/streptomycin (10,000 U/mL) at 37°C, 5% CO2.

We determined the 50% tissue culture infective dose ($TCID_{50}$) of virus stocks by means of endpoint dilution using the Reed and Muench method: In a 96-well format, we incubated 8 replicates of a 10-fold serial dilution of the stocks with cells in DMEM/F-12 containing 2% v/v FBS (2%-DMEM-FCS) and penicillin/streptomycin (10,000 U/mL) at 37°C, 5% CO_2 for 4–5 days.

Microneutralization Procedure

1) Inactivate serum samples at 56°C for 30 minutes.

2) Prepare enough diluent: DMEM/F-12 with 2% v/v FBS and penicillin/ streptomycin.

- Then, in a 96-well flat bottom plate, dilute each serum sample in duplicate, 2-fold, serially.

- Add 75uL 2%-DMEM-FCS in wells in row A (A1 to A12), and 50uL 2%-DMEM-FCS into all other wells (B1 to H12).

- Add 25uL sera into wells in row A. Mix up and down and transfer 50uL to wells in row B. Dilute serially, 2-fold and discard the last 50uL.

3) Prepare sufficient volume of 100 $TCID_{50}$ working dilution of the virus stock using the dilution media (2% DMEM-FCS).

4) Add 50uL of 100 $TCID_{50}$ working dilution to each well containing diluted samples. Total volume in each well should now be 100uL.

5) Incubate the plate(s) at 37°C, 5% CO_2 for 1 hour.

6) Prepare a control 96-well flat-bottom plate.

6.1) For back-titration, prepare 10-fold dilution series (10^{-1} to 10^{-5}) of the 100 $TCID_{50}$ virus stock working dilution.

- Add 50uL of 2% DMEM-FCS to wells from A1 to D6; then add 50uL of the 10-fold dilutions prepared above. That is, add 100 $TCID_{50}$ working dilution in first column (wells A1 to D1), 10^{-1} dilution in second column (A2 to D2), 10^{-2} in third column (A3 to D3), and so on.

6.2) Positive control: Add 50uL of the appropriate concentration or working dilution of the positive control to wells A8 to D8. Then add 50uL of 100 $TCID_{50}$ working dilution.

6.3) Negative control: Add 50uL of the appropriate concentration or working dilution of the positive control to wells A10 to D10. Then add 50uL of 100 $TCID_{50}$ working dilution.

6.4) Cell or no-virus control: Add 100uL of 2% DMEM-FCS to wells A12 to D12.

7) Suspend cells grown in flask(s) to >90% confluence in an appropriate volume of maintenance media, enough for all wells. Each well in the assay should have 100uL of cells.

- Mix well to ensure cells are evenly distributed and not clumping.

8) Transfer 100uL of cell suspension to each well in the assay plate(s) and the control plate. Aim to have 1.5-2 x 10^4 cells/well.

9) Incubate the plates at 37°C, 5% CO_2 for 4-5 days.

10) Observe daily for cytopathic effects and record the results.

Appendix Table 1 Age-specific seroprevalence and geometric mean titers of enterovirus A71, United Kingdom, 2006–2017*

	2006			2011			2017		
Age group	No. samples	Seroprevalence, % (95% CI)	GMT	No. samples	Seroprevalence (95% CI)	GMT	No. samples	Seroprevalence, % (95% CI)	GMT
<6 mo	12	66.7 (35.4–88.7)	112.4	74	74.3 (63.3–82.9)	22.5	44	63.6 (47.7– 77.2)	29.7
6–11 mo	6	33.3 (10–70)	45.2	25	44 (26.7–62.9)	33	22	18.2 (7.3–38.5)	12.3
1–5 y	92	44.6 (34.3–55.3)	90.5	46	52.2 (38.1–65.9)	313.3	82	61% (49.6–71.4)	57.3
6–10 y	42	61.9 (45.7–76)	113.5	69	69.4 (55.5–80.5)	73.8	51	66.7 (52–78.9)	172
11–20 y	110	74.6 (65.2–82.2)	151.6	76	81.6 (71.4–88.7)	67.3	98	85.7 (76.8–91.7)	64.8
21–40 y	120	80 (71.5–86.5)	42.1	75	81.3 (71.1–88.5)	51.3	149	87.3 (80.6–92)	55.3
>40 y	132	83.3 (75.6–89)	29.3	118	77.1 (69.9–83.1)	36.4	115	91.3 (84.2–95.5)	31.2

*Samples with antibody titers <8 were excluded from GMT calculation. GMT, geometric mean titer.

Appendix Table 2 Age-specific seroprevalence and geometric mean titers of coxsackievirus A6, United Kingdom, 2006–2017*

	2006			2011			2017		
Age group	No. samples	Seroprevalence, % (95% CI)	GMT	No. samples	Seroprevalence (95% CI)	GMT	No. samples	Seroprevalence, % (95% CI)	GMT
<6mo	12	75 (42.8 - 93.3)	155.2	58	65.5 (52.7–76.4)	27.7	42	66.7 (50.4–80.0)	30.1
6–11mo	5	60 (17 - 92.7)	32	22	54.5 (34.7–73.1)	55.4	22	50 (28.8–71.2)	18.7
1–5 y	87	70.1 (59.2–79.2)	100.8	39	64.1 (48.4–77.3)	69.5	82	67.1 (55.7–76.8)	228.5
6–10 y	42	73.8 (57.7–85.6)	133.8	36	77.8 (61.9–88.3)	52.5	51	76.5 (62.2–86.8)	284.8
11–20 y	110	78.2 (69.1–85.3)	121.5	67	88.1 (78.2–93.8)	63.3	97	82.5 (73.1–89.2)	84.1
21–40 y	120	89.2 (81.9–93.9)	65.7	74	83.8 (73.8–90.5)	40.5	149	89.9 (83.7–94.1)	90.5
>40 y	132	91.7 (85.2–95.6)	58	147	81.6 (74.6–87.1)	28.6	114	97.7 (79.9–92.9)	65.1

*Samples with antibody titers <8 were excluded from GMT calculation. GMT, geometric mean titer.

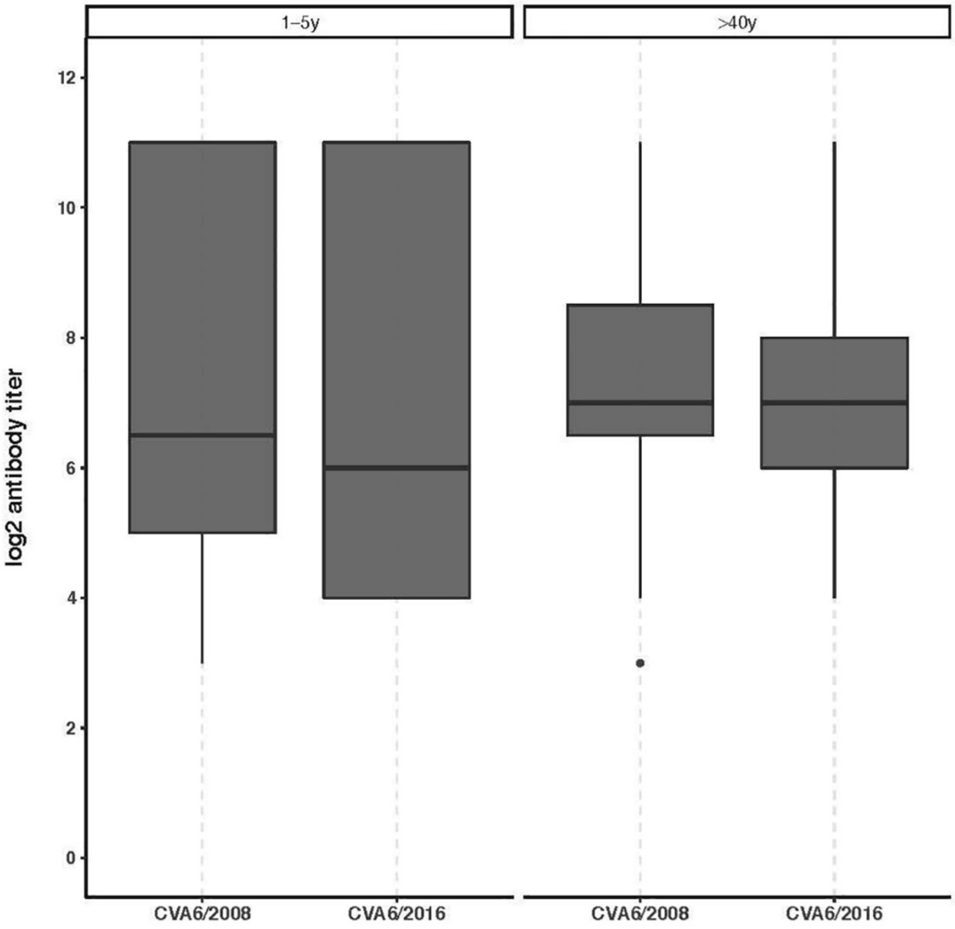

Appendix Figure 1 Boxplot showing the distribution of individual CVA6 neutralizing antibody titers against 2 CVA6 clinical strains isolated in 2008 and 2016. Black horizontal lines indicate the median and the ends of the boxes are the upper and lower quartiles. Dots show potential outliers. Whiskers indicate 95% CI. We tested 18 samples each from 2 age categories (1–5 years and >40 years). CVA6, coxsackievirus A6.

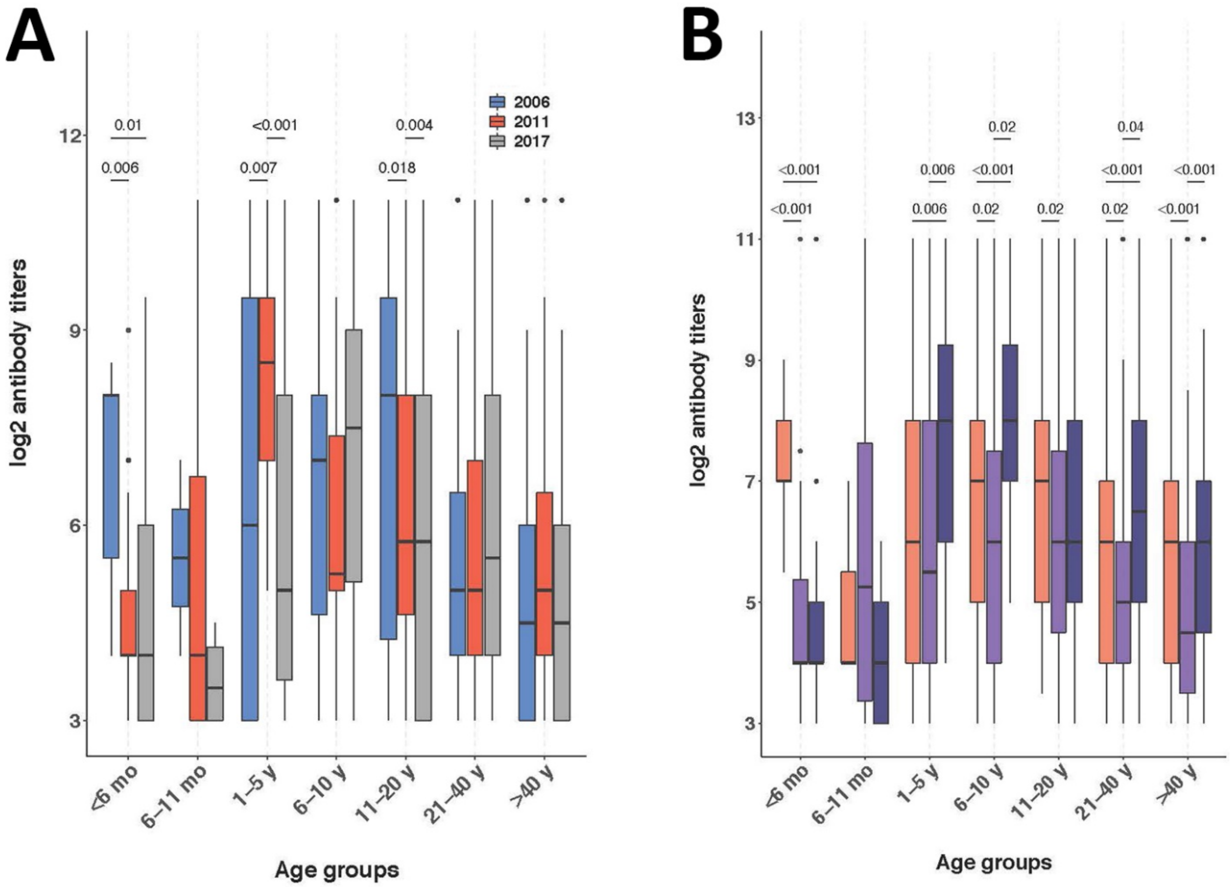

Appendix Figure 2 Boxplots showing the distribution of individual enterovirus A71 (A) and (B) coxsackievirus A6 neutralizing antibody titers for seropositive patients represented by age group. Antibody titer values are shown as log_2. Black horizontal lines indicate the median and the ends of the boxes are the upper and lower quartiles. Dots show potential outliers. Whiskers indicate 95% CI. We performed Kruskal-Wallis tests to evaluate differences in titer distributions among samples collected in the 3 years for each age group (black lines above boxplots indicate $p<0.05$).
